# Dysregulation of the calcium handling protein, CCDC47, is associated with diabetic cardiomyopathy

**DOI:** 10.1186/s13578-018-0244-0

**Published:** 2018-08-17

**Authors:** Khampaseuth Thapa, Kai Connie Wu, Aishwarya Sarma, Eric M. Grund, Angela Szeto, Armando J. Mendez, Stephane Gesta, Vivek K. Vishnudas, Niven R. Narain, Rangaprasad Sarangarajan

**Affiliations:** 1 Berg, LLC, 500 Old Connecticut Path, Bldg B (3rd Floor), Framingham, MA 01701 USA; 2 Diabetes Research Institute, University of Miami Miller School of Medicine, Miami, FL 33136 USA

**Keywords:** Diabetic cardiomyopathy, Calcium handling, CCDC47

## Abstract

**Background:**

Diabetes mellitus is associated with an increased risk in diabetic cardiomyopathy (DCM) that is distinctly not attributed to co-morbidities with other vasculature diseases. To date, while dysregulation of calcium handling is a key hallmark in cardiomyopathy, studies have been inconsistent in the types of alterations involved. In this study human cardiomyocytes were exposed to an environmental nutritional perturbation of high glucose, fatty acids, and l-carnitine to model DCM and iTRAQ-coupled LC–MS/MS proteomic analysis was used to capture proteins affected by the perturbation. The proteins captured were then compared to proteins currently annotated in the cardiovascular disease (CVD) gene ontology (GO) database to identify proteins not previously described as being related to CVD. Subsequently, GO analysis for calcium regulating proteins and endoplasmic/sarcoplasmic reticulum (ER/SR) associated proteins was carried out.

**Results:**

Here, we identified CCDC47 (calumin) as a unique calcium regulating protein altered in our in vitro nutritional perturbation model. The cellular and functional role of CCDC47 was then assessed in rat cardiomyocytes. In rat H9C2 myocytes, overexpression of CCDC47 resulted in increase in ionomycin-induced calcium release and reuptake. Of interest, in a diet-induced obese (DIO) rat model of DCM, CCDC47 mRNA expression was increased in the atrium and ventricle of the heart, but CCDC47 protein expression was significantly increased only in the atrium of DIO rats compared to lean control rats. Notably, no changes in ANP, BNP, or β-MHC were observed between DIO rats and lean control rats.

**Conclusions:**

Together, our in vitro and in vivo studies demonstrate that CCDC47 is a unique calcium regulating protein that is associated with early onset hypertrophic cardiomyopathy.

**Electronic supplementary material:**

The online version of this article (10.1186/s13578-018-0244-0) contains supplementary material, which is available to authorized users.

## Background

Diabetes mellitus is associated with an increased risk in diabetic cardiomyopathy (DCM) that is distinctly not attributed to co-morbidities with other vasculature diseases, which include coronary artery disease (CAD) or hypertension [1, 2]. It is characterized by early-onset diastolic dysfunction followed by late-onset systolic dysfunction, which is thought to be mediated by cardiac remodeling and hypertrophy [3]. Studies to date have demonstrated that multiple cellular and molecular dysfunctions contribute to the pathogenesis of DCM. For example, increased free fatty acids, hyperglycemia, and inflammation have been shown to promote endoplasmic reticulum (ER) stress and the unfolded protein response (UPR), which has been implicated in cellular damage and fibrosis associated with DCM [4]. Energy stress, shift in metabolic fuel sources, and dysregulation of metabolic signaling have also been implicated as contributing mechanisms to the pathogenesis of DCM [2]. Lastly, and of particular interest, is that dysregulation of calcium (Ca^2+^) handling has also been reported as playing a key role in development of DCM [2, 3, 5].

Intracellular Ca^2+^ plays a major role in cardiomyocyte function via its regulation of signaling cascades that can affect protein activity and downstream gene expression and notably via its role in excitation-contraction coupling (ECC). Contraction of cardiomyocytes is mediated by Ca^2+^ entry via l-type Ca^2+^ ion channels, triggering ryanodine receptor (RyR)-induced release of calcium stores from the ER or sarcoplasmic reticulum (SR), which in turn results in a surge in intracellular Ca^2+^ [Ca^2+^]_I_ that binds to and activates the myofibril protein, troponin C [6–8]. In contrast, cardiomyocyte relaxation is mediated by the reduction of intracellular Ca^2+^ via release of Ca^2+^ from troponin and Ca^2+^ reuptake by SR Ca^2+^ ATPase (SERCA), sarcolemmal Na/Ca^2+^ exchange (NCX), sacrolemmal Ca^2+^-ATPase, and/or mitochondrial Ca^2+^ uniport [9]. Of interest, in in vivo rodent models of diabetes alterations in expression and/or a number of proteins affecting the ECC have been reported, however, with inconsistent findings. For example, Pereira et al. found that cardiac contractile function was impaired, but this was not associated with hypertrophy in db/db mice [10]. In this same study, db/db mice had reduced levels of SR Ca^2+^, decreased protein expression of RyR, and decreased activity of SERCA, but SERCA protein levels were not altered [10]. In contrast, Belke et al. did not find alterations in cardiac RyR protein expression in db/db mice compared to control mice, but there was a slight nonsignificant decrease in SERCA2 protein expression and an increase in phospholamban (PLN) phosphorylation [11]. In addition, while Pereira et al. found no change in SERCA2 in db/db mice, reduction in SERCA2 protein levels have been demonstrated in a severe diabetes rat model, the Otsuka Long Evans Tokushima Fatty rats [12], and reduction in mRNA levels have been reported in streptozotocin-diabetic rats [13]. Moreover, in contrast to the models mentioned above, in an early stage type 2 diabetes model (ZDF rat) SERCA mRNA expression was found to be increased, while PLN mRNA was reduced, and these alterations were associated with an increase in SR Ca^2+^ load [14]. The inconsistent findings could be attributed to differences in models and/or stage of disease.

Given the inconsistent findings presented above, the present study was designed to identify unique calcium regulating and or ER/SR related proteins that may be associated with DCM in cardiomyocytes. In this study human cardiomyocytes were exposed to an environmental nutritional perturbation of high glucose, fatty acids, and l-carnitine and iTRAQ-based quantitative proteomic analysis was used to assess proteins affected by the perturbation. The proteins captured were then compared to proteins currently annotated in the cardiovascular disease (CVD) gene ontology (GO) database to identify proteins not previously described as being related to CVD. Subsequently, GO analysis for calcium regulating proteins and ER/SR associated proteins was carried out. Here, we identified CCDC47 (calumin) as a unique protein and in vitro and in vivo studies were performed to validate our findings and characterize the cellular function of CCDC47.

## Results

### Identification of unique proteins associated with calcium regulation in an in vitro nutritional model of cardiomyopathy

**Fig. 1:**
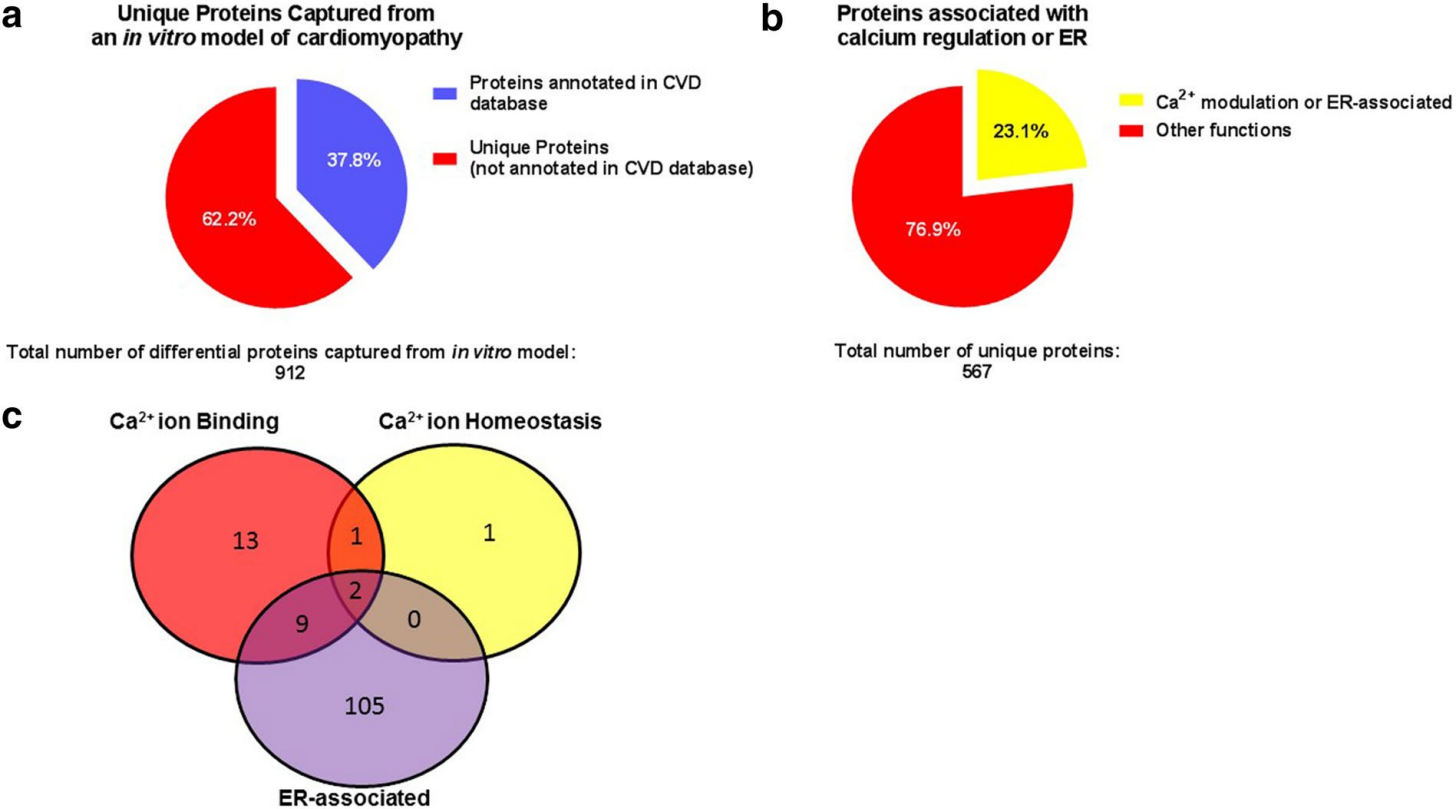
In vitro model of cardiomyopathy identifies unique calcium regulating proteins. **a** Percentage of proteins in Uniprot’s cardiovascular disease (CVD) database that were captured from the in vitro model. **b** Percentage of the unique proteins that are associated with calcium regulation or endoplasmic reticulum/sarcoplasmic reticulum (ER/SR). **c** Venn diagram illustrates the number of proteins identified in each gene ontology category and their overlap as annotated in Uniprot’s *Homo sapiens* database

DCM is associated with increased circulating free fatty acids, hyperglycemia and inflammation, which contributes to ER stress, shift in cellular metabolism, disruption of intracellular signaling and calcium handling [3]. Thus, we examined the effect of an in vitro nutritional perturbation on cardiomyocytes to identify proteins affected by nutrients known to disrupt cellular metabolic processes, promote oxidative stress and affect cardiovascular health. Human cardiomyocytes of donors with cardiomyopathy were exposed to either high glucose, free fatty acids (linoleic acid and oleic acid), and l-carnitine or control media containing normal glucose (5 mM) for 6 h. Cell lysates were then subjected to proteomic analysis. A total of 2669–3217 proteins were captured across donor samples, but 1283 proteins were matched among all samples. Of these, 912 proteins were significantly altered due to treatment with high glucose and free fatty acids. The list of 912 proteins were then compared to proteins annotated in the UniProt-GOA database, which contains over 4000 proteins. The in vitro nutritional model of cardiomyopathy identified 8.1% (345 out of 4222) of the proteins currently annotated in the UnitPro-GOA cardiovascular disease (CVD) database. Furthermore, of the 912 proteins that were differentially affected by the nutritional perturbation in the in vitro model, 62.2% (567 out of 912) are unique, as they have not yet been identified as being associated with CVD and/or annotated in the UniProt-GOA CVD database (Fig. 1a). To confirm that the likelihood of proteins captured in the CVD database are higher than being captured in a non-CVD database the 912 differential proteins were compared to the UniProt-GOA renal disease database. The UniProt-GOA renal disease database contains a total of 3713 proteins, however, only 1591 proteins (42.8%) are identified as human. Comparison of the proteins captured from the in vitro cardiomyopathy model (912 total) to the human proteins listed in the renal disease database (1591) identified 123 matches between the two databases. Moreover, 86.5% (789 out of the 912) of the proteins captured in the in vitro cardiomyopathy model are unique, i.e. they are not related to renal disease and/or annotated in the renal disease database (Additional file 1: Figure S1). Thus, these results highlight the ability of the in vitro nutritional perturbation to model cardiovascular disease and enables the identification of unique proteins affected by nutritional perturbations in cardiomyocytes.

Next, we sought to identify proteins that are localized in the ER/SR that may have a putative role in calcium regulation (binding or homeostasis) by using gene ontology (GO) analysis of the unique proteins captured. GO analysis was performed by extracting all currently annotated GO terms (GO identification [ID]) for each of the 567 unique proteins (as listed in the UnitPro *Homo sapiens* database) followed by matching all GO IDs for each protein to GO IDs for ER localization, calcium ion homeostasis, calcium ion binding, and other relevant calcium related properties (Fig. 1b). This analysis revealed that of the 567 unique proteins differentially affected by nutritional perturbation 4 proteins play a role in calcium ion homeostasis, 25 proteins are involved in calcium ion binding, and 116 proteins are localized in the ER (Additional file 2: Table S1). The Venn diagram illustrates the 2 unique proteins as belonging to all 3 categories (annexin A7 [P20073] and coiled–coiled domain binding protein 47, coil–coiled domain 47, CCDC47 [Q96A33]), 9 unique proteins as localized in the ER and involved in calcium ion binding (peptidyl-prolyl cis–trans isomerase FKBP9 [O95302], gelsolin [P06396], 78 kDa glucose-regulated protein [P11021], glucosidase 2 subunit beta [P14314], calnexin [P27824], reticulocalbin-2 [Q14257], reticulocalbin-1 [Q15293], peptidyl-prolyl cis–trans isomerase FKBP10 [Q96AY3], reticulocalbin-3 [Q96D15]), and 1 unique protein involved in calcium ion homeostasis and calcium ion binding (translationally-controlled tumor protein [P13693]) (Fig. 1c).

**Fig. 2:**
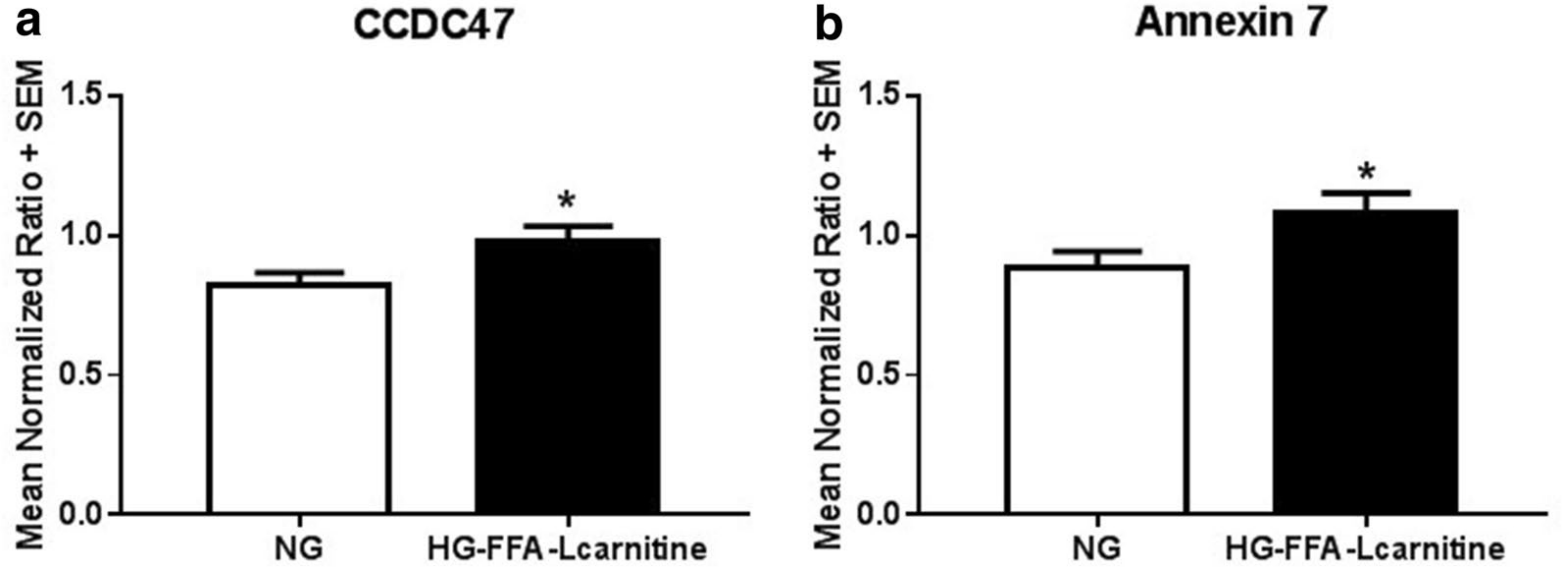
Differentials of the two proteins identified as being localized in the endoplasmic reticulum, regulation of calcium ion homeostasis and calcium ion binding. Primary cardiomyocytes were plated overnight then exposed to high glucose (20 mM) and free fatty acids (oleic acid 3.33 μM, linoleic acid 3.33 μM, and l-carnitine 1 mM or kept under normoglycemic conditions for 6 h prior to harvesting for quantitative proteomic analysis by stable isotope labeling using 8-plex iTRAQ coupled to 2D-LC MALDI MS/MS. All experimental samples were then normalized to a pooled universal reference sample labeled with 113 reagent (as described in “Methods”). **a** CCDC47 and **b** Annexin 7. *p < 0.05 compared to cardiomyocytes under normal glucose (control). Bar graphs indicate mean normalized ratio + standard error of n = 5 independent biological samples

The two proteins that were identified as belonging into all categories of interest showed significant upregulation in response to treatment with HG, FFA, and l-carnitine (Fig. 2). Annexin A7 is a protein that belongs to a family of annexins, which are Ca^2+^ and phospholipid ion binding proteins. Annexins have been proposed to play a role in calcium handling in cardiomyopathy [15]. In addition, prior research has demonstrated that annexin 7 is involved in excitation–contraction coupling possibly via regulation of calcium homeostasis [16]. Very little is known about CCDC47 and to the best of our knowledge there are no studies linking CCDC47 to cardiomyopathy. Thus, we chose to further investigate CCDC47 and its association with cardiomyopathy.

### Functional characterization of CCDC47 in cardiomyocytes

**Fig. 3:**
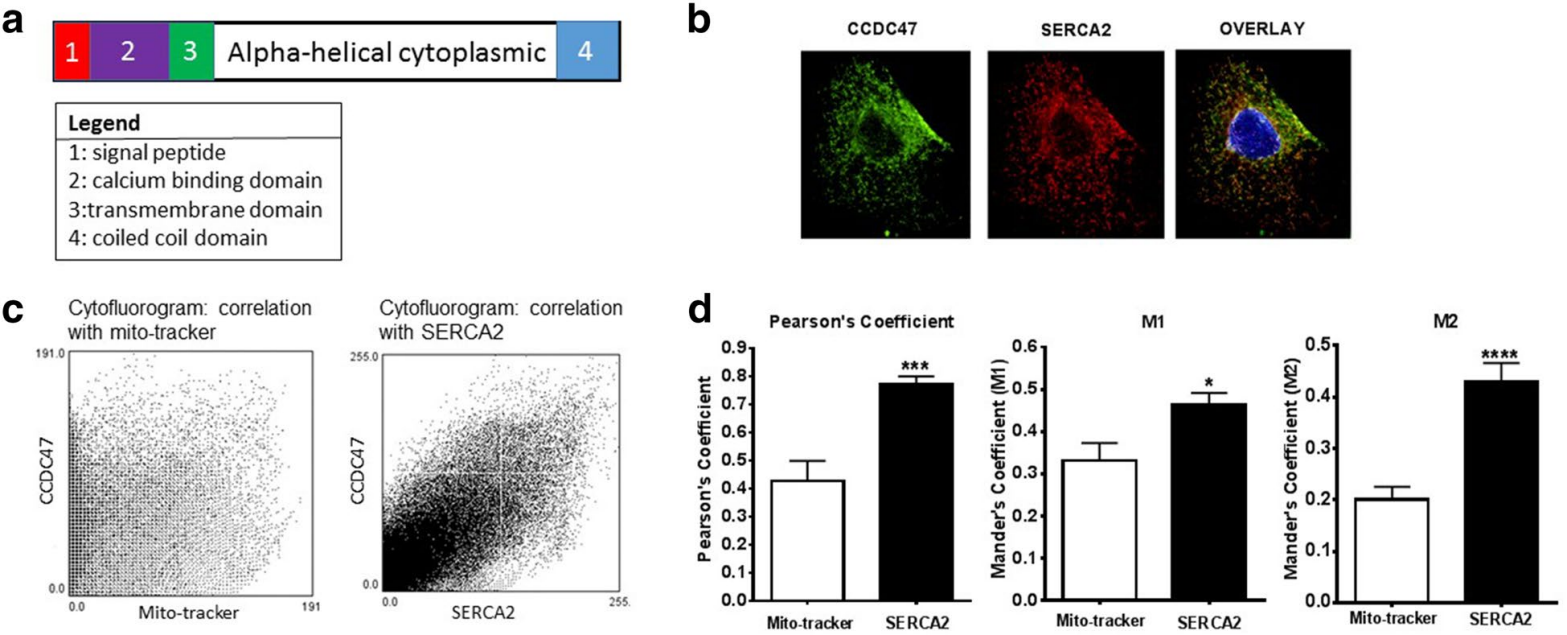
CCDC47 is localized in the endoplasmic reticulum (ER) and regulates calcium flux in rat H9C2 cardiomycotyes. **a** Schematic of CCDC47 as a transmembrane protein that contains an N-terminal ER signaling peptide and calcium binding domain. **b** Immunohistochemical staining of CCDC47 revealed co-localization with the ER marker SERCA2 in H9C2 myocytes. Green channel indicates CCDC47, red channel indicates SERCA2, and merged image indicates co-localization in yellow. Nuclear staining (blue) was performed with DAPI. **c** Cytofluorogram depicts degree of overlay between CCDC47 and mitochondria labeled with mito-tracker (left panel) and overlay between CCDC47 and SERCA2 (right panel). **d** Quantification of degree of overlay using Pearson’s coefficient, M1, and M2 values. All three measurements revealed significant co-localization of CCDC47 with SERC2A compared to mito-tracker. Bar graphs indicate mean + standard error of n = 3–4 independent biological replicates. *p < 0.05 and ***p < 0.001 compared to mito-tracker

CCDC47 (also known as calumin) is a 483 amino acid single transmembrane protein. Structural domain analysis indicates that it contains an ER signaling peptide localized at the N-term, a calcium binding domain, transmembrane domain, and cytosolic domain localized at the C-term (see Fig. 3a). Based on this analysis CCDC47 is predicted to play a role in ER-regulated calcium handling and homeostasis. Indeed, prior studies have shown that CCDC47 binds Ca^2+^ and regulates calcium homeostasis in mouse embryonic fibroblasts [17]. To confirm this in cardiomyocytes, subcellular localization of CCDC47 was examined using immunocytochemistry and flow cytometry. Immunocytochemistry and flow cytometric studies in H9C2 rat cardiomyocytes demonstrate expression and distribution of CCDC47 in the ER as indicated by co-localization with sarco/endoplasmic reticulum Ca^2+^ ATPase (SERCA2) (Fig. 3b–d). Co-localization of CCDC47 with SERCA2 was significantly higher than that with mitochondria-tracker (Pearson coefficient 0.428 ± 0.07 vs. 0.771 ± 0.03, *p* < 0.001; M1 = 0.3316 ± 0.04 vs. 0.4641 ± 0.03, *p* < 0.05; M2 = 0.2008 ± 0.03 vs. 0.4295 ± 0.04, *p* < 0.001 for mitochondria-tracker vs. SERCA2, respectively) (Fig. 3d), further supporting its localization at the SR.

### CCDC47 regulates intracellular calcium, which may regulate expression of CCDC47 in H9C2 cardiomyocytes

**Fig. 4:**
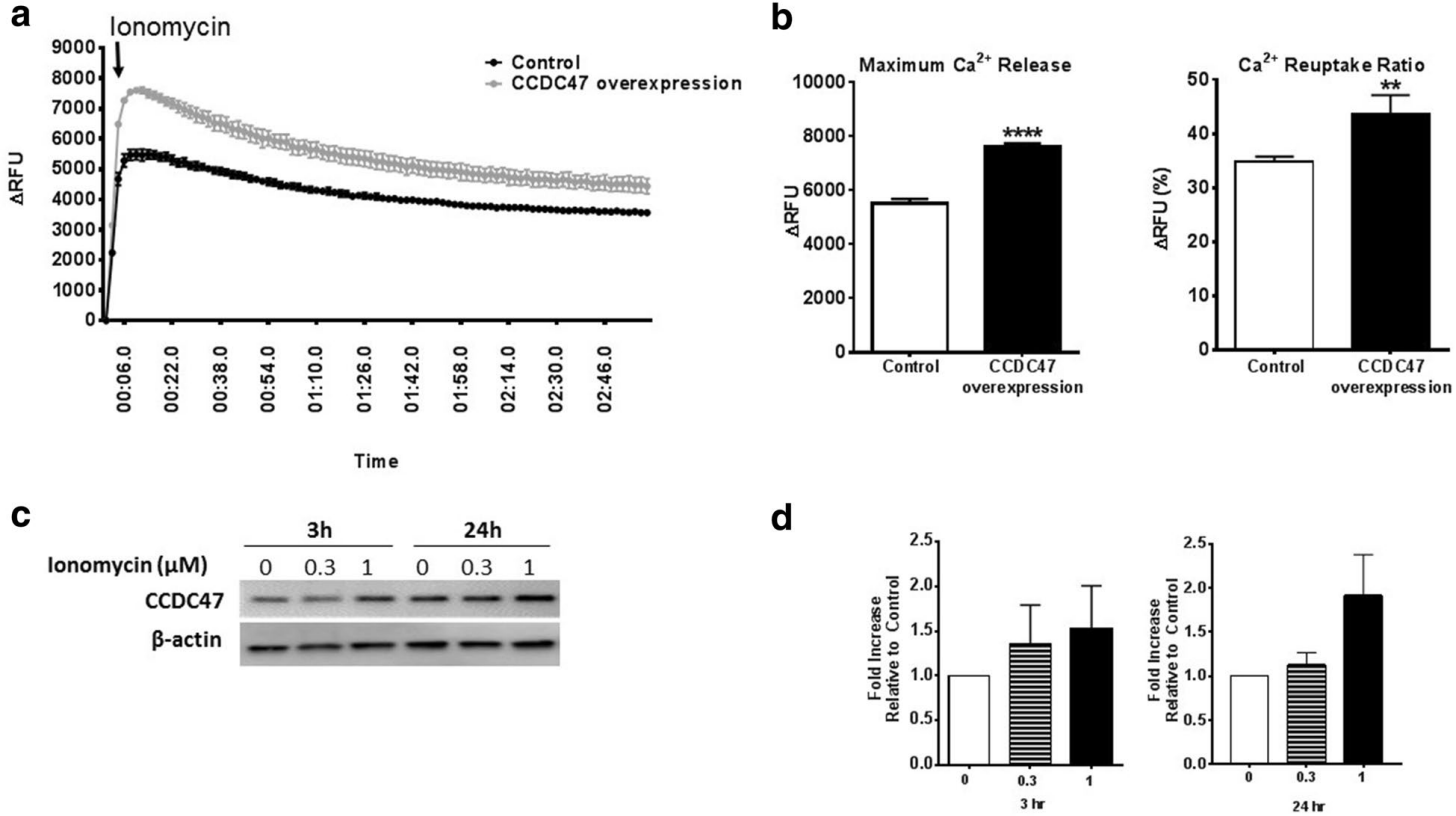
CCDC47 regulates calcium flux and its expression may be regulated by intracellular calcium levels in rat H9C2 cardiomyocytes. **a** CCDC47 overexpressed in rat H9C2 myocytes in the presence of 2 mM calcium show increased cytosolic calcium release induced by 1 µM ionomycin as represented over time. **b** Maximum calcium release (left graph) and calcium reuptake (right graph) was also higher in cells that overexpressed CCDC47. Bar graphs indicate mean + standard error of n = 3 independent biological replicates. **p < 0.01 and ****p < 0.0001 compared to control (empty vector). **c**, **d** Time- and concentration-dependent effect of ionomycin on CCDC47 protein expression. **c** Representative Western blot and **d** densitometric analysis. Immunoreactive band intensity for all Western blot data was normalized to β-actin and plotted relative to control. Bar graphs indicate mean + standard error of n = 3 independent biological replicates

In mouse embryonic fibroblasts, knockdown of CCDC47 was associated with impaired calcium signaling [17]. Thus, we examined the relationship between intracellular calcium levels and CCDC47 in excitable cardiomyocytes. H9C2 myocytes were transfected with either empty vector control or CCDC47 for 24 h (Additional file 3: Figure S2) and calcium flux was measured using a fluoroforte calcium assay kit. In the presence of 2 mM Ca^2+^ myocytes were stimulated with ionomycin to mobilize calcium stores [18]. As shown in Fig. 4, ionomycin (1 µM) induced a robust increase in release of Ca^2+^ stores. Notably, in cells overexpressing CCDC47 maximal release was significantly greater than control cells. Reuptake, as measured by determining the amount of Ca^2+^ loss after maximal response relative to the balance (final response), was also greater in cells that overexpressed CCDC47 compared to control cells (Fig. 4b). Lastly, the effects of increasing intracellular calcium levels with ionomycin at different time points and concentrations on CCDC47 protein expression was examined in non-transfected cardiomyocytes (Fig. 4c, d). Ionomycin induced an increase in CCDC47 in a dose-dependent manner, however, this response did not reach statistical significance at any time or dose administered. Together, these data support a role for CCDC47 in regulation of intracellular calcium release and storage and suggests that ionomycin could potentially induce an increase in CCDC47 protein expression.

### Characterization of diet-induced obesity as a model for early onset hypertrophic cardiomyopathy

**Fig. 5:**
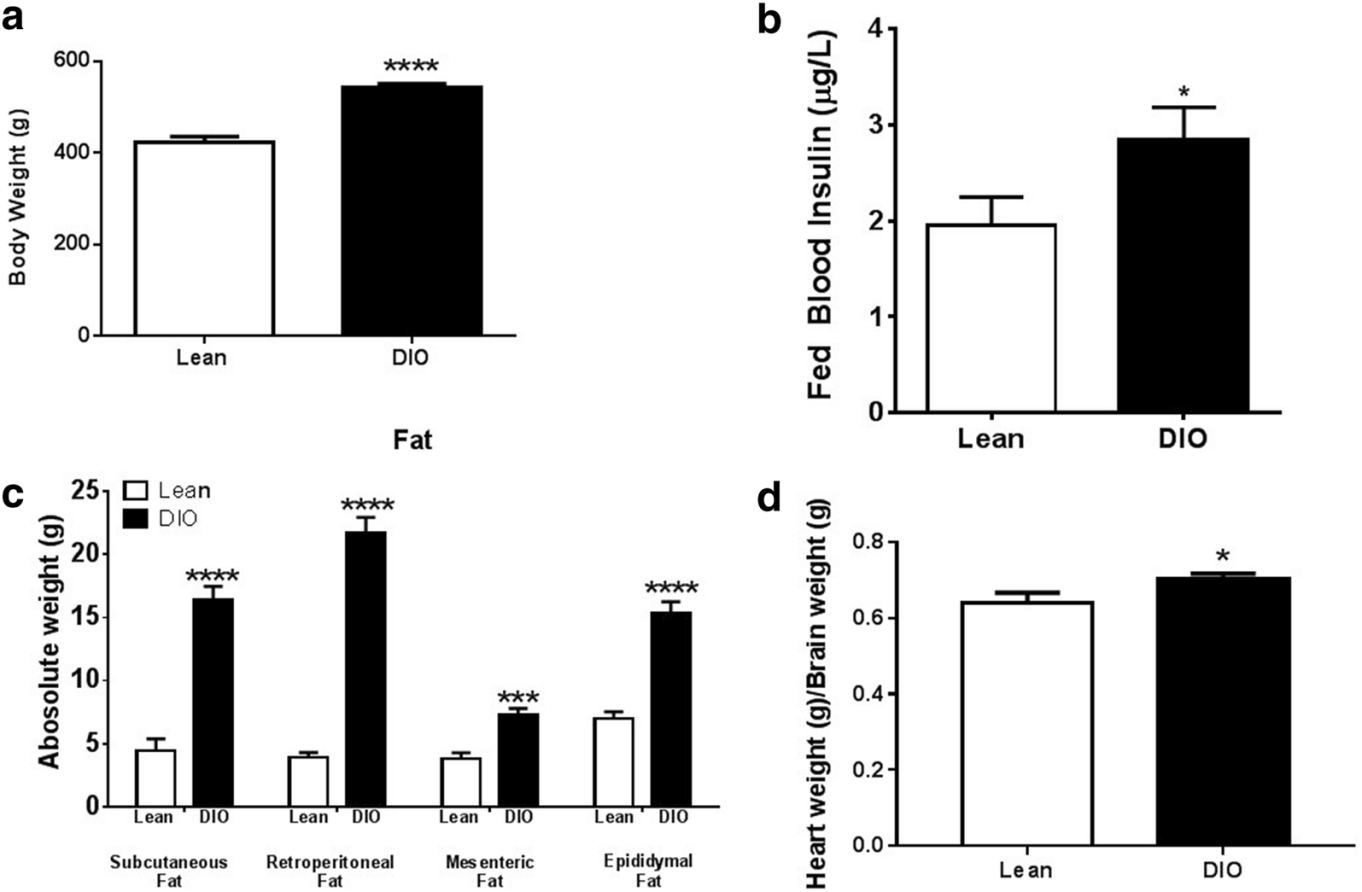
Characteristic phenotype of a diet-induced obesity (DIO) rat model of cardiac hypertrophy. **a** Male Wistar rats (4 weeks old) that were maintained on a high fat diet for 6 weigh more than control rats fed a lean diet. **b** Insulin levels of rats measured at 20 weeks. White bars represent lean rats (n = 7) and black bars represent DIO rats (n = 8). **c** Fat content from subcutaneous abdomen, retroperitoneal and mesenteric compartments is increased in DIO rats compared to lean rats. **d** Ratio of heart weight/brain weight is increased in DIO rats compared to control rats. Bar graphs indicate mean + standard error. *p < 0.05, ***p < 0.001 and ****p < 0.0001 compared to lean control rats

Rats fed a high fat diet for 6 months (a model for diet-induced obesity) develop cardiomyopathy [19]. In contrast, rats fed a moderate fat diet for 12 weeks do not have significant cardiac abnormalities, but this treatment paradigm is sufficient to induce alterations in renal function and blood pressure [20]. Thus, in these studies we used a treatment paradigm that would produce diet-induced obese rats with very early onset hypertrophic cardiomyopathy. Specifically, rats were fed either a lean diet (control) or high fat diet (diet-induced obese, DIO) for 6 weeks beginning at 4 weeks of age. Cardiac abnormalities and alterations in CCDC47 were examined. First, the phenotypic characteristics of rats that were fed a lean or high fat diet for 6 weeks were evaluated. As shown in Fig. 5a, DIO rats weighed significantly more than lean controls. DIO rats showed an increase in insulin levels compared to lean rats (*p* < 0.05, Fig. 5b). Not surprisingly, absolute weights of subcutaneous, retroperitoneal, mesenteric and epididymal fat were significantly increased in DIO rats compared to lean rats (Fig. 5c). In addition, there was a significant increase in heart weights from DIO rats compared to lean rats (Fig. 5d). Together, these results suggest that rats fed a high fat diet for 6 weeks is an appropriate model for cardiomyopathy due to the increase in body weight, fat content, and heart weight.

### Biomarkers for cardiomyopathy are not altered in DIO rats

**Fig. 6:**
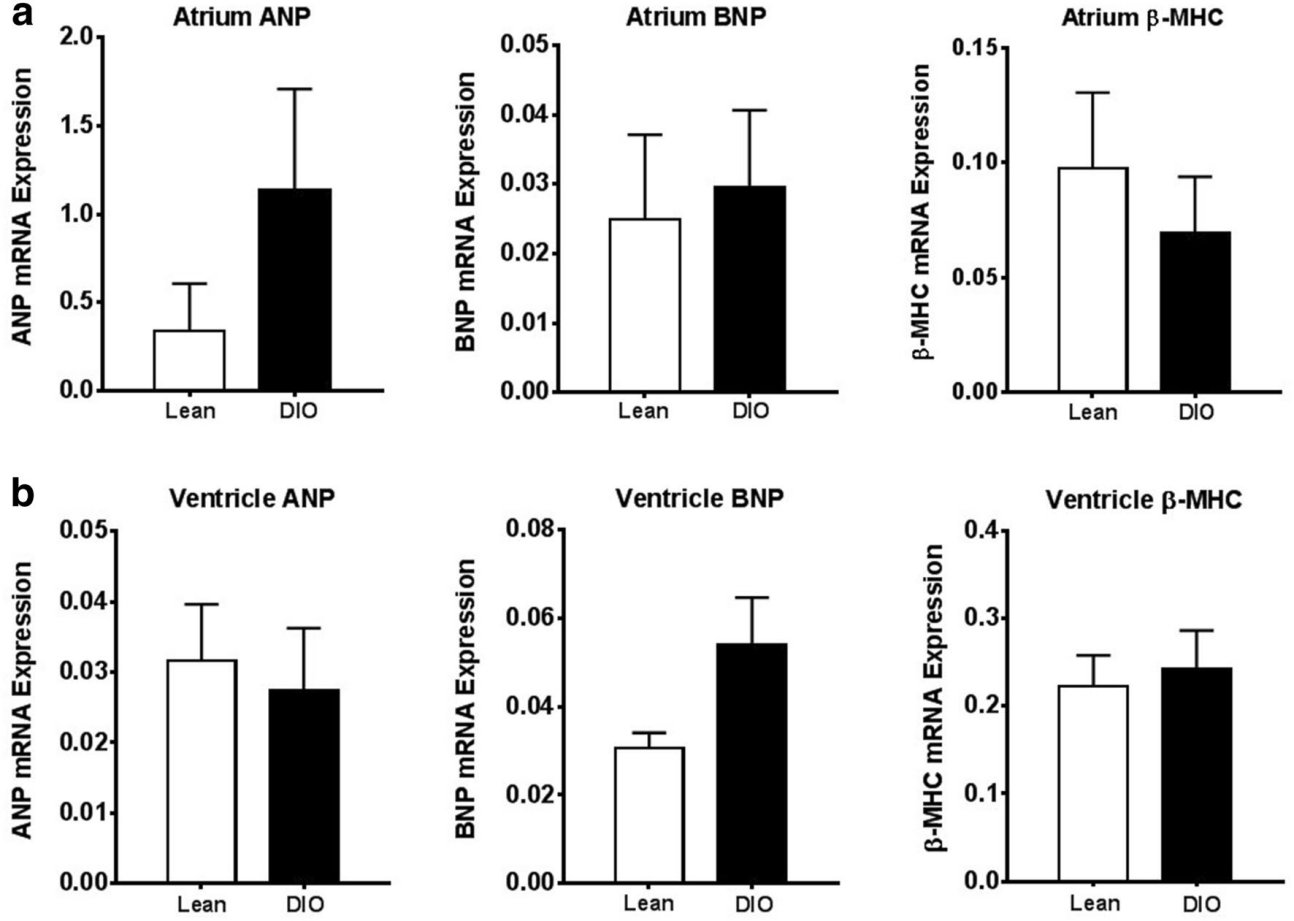
Biomarkers for cardiac hypertrophy are not significantly altered in atrium or ventricle of hearts from DIO rats with early-onset cardiac hypertrophy. **a** mRNA expression of atrial natriuretic peptide (ANP), brain natriuretic peptide (BNP), and myosin heavy chain beta (β-MHC) in the atrium of heart from lean rats and DIO rats. **b** mRNA expression of ANP, BNP, and β-MHC in the ventricle of heart from lean rats and DIO rats. Bar graphs indicate mean + standard error

Next, whether alterations in biomarkers known to play a role in cardiomyopathy could be detected in the present animal model was examined. Here, mRNA expression levels of atrial natriuretic peptide (ANP), brain natriuretic peptide (BNP), and myosin heavy chain beta (β-MHC) from the atrium and ventricle regions of the heart were measured by real-time quantitative PCR. Notably, there were no statistically significant differences between DIO rats and lean rats in ANP, BNP, or β-MHC from the atrium (Fig. 6a) or ventricles (Fig. 6b).

### CCDC47 mRNA and protein expression is increased in a model of cardiomyopathy

**Fig. 7:**
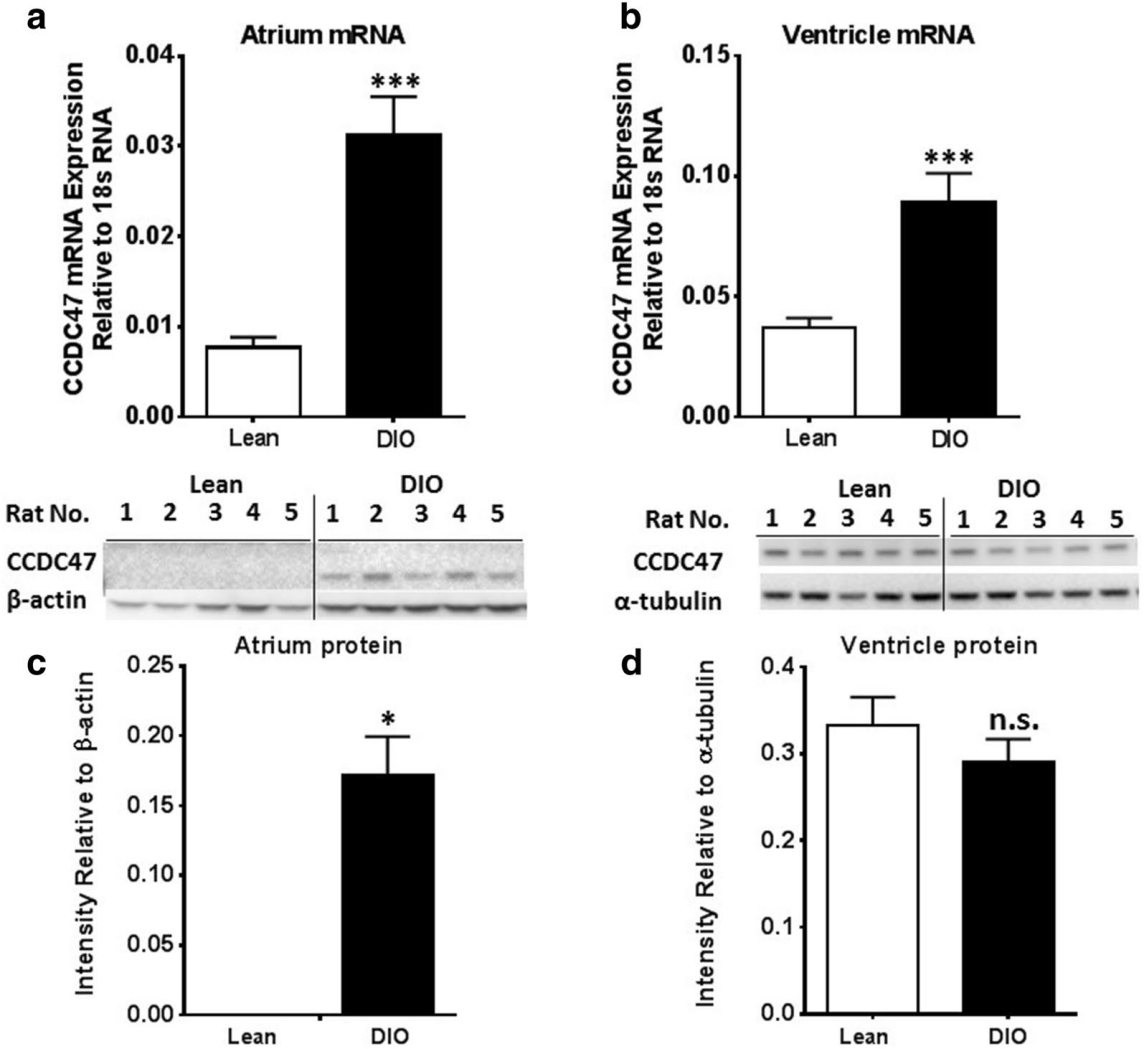
CCDC47 mRNA and protein expression is increased in hearts of DIO rats. **a** CCDC47 mRNA levels in the atrium is increased in DIO rats (n = 4) compared to lean rats (n = 5). **b** CCDC47 mRNA levels in the ventricle is increased in DIO rats (n = 5) compared to lean rats (n = 7). **c** Representative Western blot of protein levels of CCDC47 in atrium of lean (n = 5) and DIO rats (n = 5) (top panel) and quantification of immunoreactive bands normalized to β-actin (bottom panel). Each lane represents an individual animal. **d** Representative Western blot of protein levels of CCDC47 in ventricle of lean and DIO rats. Quantification of immunoreactive bands normalized to α-tubulin were obtained from ventricle of lean (n = 9) and DIO (n = 8) rats. Each lane represents an individual animal. Bar graphs indicate mean + standard error. *p < 0.05 compared to lean control rats; ***p < 0.001 compared to lean control rats; *ns* non-significant difference compared to lean control rats

Disruption in calcium homeostasis occurs very early in the pathogenesis of hypertrophy cardiomyopathy [21]. Given the in vitro findings presented here that support a role for CCDC47 in regulation of calcium handling and alterations in intracellular calcium induces CCDC47 expression, CCDC47 expression was examined. As shown in Fig. 7, CCDC47 mRNA and protein levels are significantly altered in the hearts of DIO rats compared to lean rats. Specifically, in the atrium there were significant increases in CCDC47 mRNA and protein expression in DIO rats compared to lean rats (Fig. 7a, c). In the ventricle, CCDC47 mRNA expression in DIO rats was significantly increased compared to lean rats, but there was no statistical difference in protein levels between groups (Fig. 7b, d). These data demonstrate CCDC47 expression is altered in diet-induced obesity in the present model of cardiomyopathy and suggests that CCDC47 is altered in the early pathogenesis of cardiomyopathy.

## Discussion

### In vitro model of diabetic cardiomyopathy identifies a number of unique proteins involved in calcium regulation

In the present study we used a proteomic approach to identify proteins altered by prolonged nutritional perturbations in vitro to model cardiomyopathy associated with diabetes. However, we focused on examining proteins involved in calcium regulation and those associated with the ER or SR as calcium plays a key role in modulating ECC of cardiomyocytes [9, 22] and as an upstream signaling molecule it also functions as an inductor of gene expression associated with ‘remodeling’ and promoting maladaptive cardiac hypertrophy [2]. In the present study we found that nearly 38% of the proteins that were differentially expressed in our in vitro model have previously been identified as being associated with cardiovascular disease, while 62% were unique. Of the unique proteins captured in the present model, 23% of the differential proteins are associated with calcium regulation and/or the ER or SR. CCDC47 (calumin) was identified as a unique protein that has not previously been associated with cardiomyopathy.

### CCDC47 regulates calcium homeostasis in rat myocytes

Prior studies have shown that CCDC47 is a high capacity Ca^2+^ binding protein with moderate affinity (K_d_ = ~ 0.75 mM) for Ca^2+^ in mouse embryonic fibroblasts [17]. Knockdown of CCDC47 resulted in impaired calcium signaling, in particular store-operated Ca^2+^ entry (SOCE), which notably was not associated with alterations in expression of other calcium handling proteins including SERCA, CNX, and CRT [17]. CCDC47 shares structural homology to stromal interaction molecule 1 (STIM1), a calcium sensor that is key regulator of SOCE [23]. Like STIM1, CCDC47 is a single transmembrane binding protein that contains a calcium binding and coiled–coil domain [24]. In non-excitable cells, the coiled–coil domain of STIM is required for homodimerization and promoting conformational changes that mediate the ability for STIM to translocate to the plasma membrane at the ER-plasma membrane junction to in turn regulate the Ca^2+^ release-activated Ca^2+^ channel (CRAC), orai1 [25, 26]. Thus, given the structural similarities it has been proposed that CCDC47 shares similar functional properties to STIM1 [24]. Our study supports this possibility as overexpression of CCDC47 in cardiomyocytes potentiated ionomycin-induced increase in intracellular calcium, affecting both release and uptake.

In the present study we also demonstrate that intracellular calcium signaling may affect expression of CCDC47. Here, we note that significant increases in CCDC47 expression after ionomycin was not statistically significant, even after 24 h of treatment at a high dose of 1 μM, which is consistent with a previous report demonstrating that ionomycin was unable to promote increase in cardiomyocyte hypertrophy [27]. Thus, it is possible that in a chronic or stable cellular model of cardiomyopathy calcium signaling may in turn affect CCDC47 expression. Nevertheless, these data indicate a role of CCC47 in calcium regulation in normal rat myocytes.

### CCDC47 upregulation in a DIO model of cardiomyopathy

While the effect of diabetes on left ventricular hypertrophy has been studied [2], less is known about alterations in the atrium [28]. Disruption in calcium homeostasis occurs very early in the pathogenesis of cardiomyopathy [21] and as discussed previously, these alterations may contribute early on in the development of DCM. In the present study in a DIO model CCDC47 mRNA expression was increased in atrium and ventricles, but protein levels were only significantly elevated in the atrium. This compartment specific phenomenon is not surprising given that differential expression of proteins has been reported in normal fetal heart [29]. In fact, it has been shown that the mRNA expression of BNP is increased in the atrium, but not ventricle, of streptozotocin-induced diabetic pigs [30]. Thus, it is not surprising that alterations in proteins can differ between chambers in various disease states, not just DCM. For example, this phenomenon has been observed with atrial fibrillation [30], during experimental heart failure [31, 32], and in children with congenital heart disease [33]. Alternatively, this compartment differential suggests that CCDC47 protein is upregulated in the atrium earlier, perhaps more rapidly, than in the ventricle. It is possible that CCDC47 protein levels would be increased in the ventricle if we assessed the levels at a later time point. Another possibility is that the early increase in CCDC47 protein levels in the atrium may play a role in atrial fibrillation. Indeed, prior studies have demonstrated that alterations in calcium handling and disruption in SR function is associated with atrial fibrillation [34, 35]. Of interest, increased methylation of an upstream region in the CCDC47 gene was found in blood samples from patients with coronary artery disease [36]. It must be noted that in model used in the present study the well-described biomarkers ANP, BNP, and β-MHC [37] were not significantly altered. Cardiac ‘remodeling’ and hypertrophy associated with alterations in ANP, BNP, and β-MHC require the induction of reprogramming of genes. This suggests the possibility that disruptions calcium handling affects CCDC47 expression more rapidly than genetic reprogramming and implicate that alterations in calcium regulating proteins that occur upstream of gene transcription may lead to the identification of proteins involved in the early process/stage of cardiomyopathy. Indeed, as increase in plasma glucose levels appear early in diabetes, even as early as in the pre-diabetic stage, the hyperglycemic state can promote the glycosylation of proteins and affect protein activity. In line with this, recent studies have shown that O-linked *N*-acetylglucosamine (O-GlcNAc) of CaMKII is increased in the heart and brain of diabetic humans and rats and in cardiomyocytes is associated with increased spontaneous SR Ca^2+^ release [38]. It has thus been proposed that the alterations in increased SR Ca^2+^ leak may be involved in the development of DCM [3]. Of interest, CCDC47 is predicted to have an N-linked glycosylation site at Asn178. However, it is unknown whether glycosylation of CCDC47 could affect its molecular function.

## Conclusions

In summary, using an in vitro nutritional model of cardiomyopathy we identified a number of unique proteins that have not previously been found to be associated with cardiovascular disease. To the best of our knowledge this is the first report to characterize CCDC47 in excitable cells, i.e., cardiomyocytes. Moreover, this is the first report to identify altered expression of CCDC47 in a DIO model of cardiomyopathy. Here, we have shown that CCDC47 regulates calcium homeostasis. Together, these studies demonstrate that dysregulation of CCDC47 may play a role in diet-induced cardiomyopathy.

## Methods

### Cell lines

Primary cardiomyocytes from 5 different donors (Mean age = 55, range 50–61, Caucasian) with cardiomyopathy were obtained from Promocell (Heidelberg, Germany) and maintained in myocyte growth media (Promocell, Heidelberg, Germany) at 37 °C. For perturbation experiments cells were plated overnight (24 h) then incubated in media with high glucose (20 mM) and free fatty acids [oleic acid 3.33 μM (Sigma), linoleic acid 3.33 μM (Sigma), and l-carnitine 1 mM (Sigma)] for 6 h prior to harvesting for proteomic analysis. Rat embryonic cardiomyocytes [H9C2(2–1)] were obtained from ATCC (ATCC^®^ CRL-1446™) and maintained in DMEM growth media (Lonza-12-604F) supplemented with 10% fetal bovine serum (Gibco) at 37 °C. H9C2 myocytes were seeded 24 h prior to differentiation into myotubes by reducing serum concentration to 1% serum within the culture media and incubated for 48 h.

### Animals

Male Wistar rats were obtained from Charles River Laboratories (Wilmington, MA) at 4 weeks of age, housed 2 per cage at 22 °C on a 12:12 h day–night cycle, and given water and fed high-fat diet (Research Diets, St. Louis, MO; 60 kcal % fat, 20 kcal % protein, and 20 kcal % carbohydrate) ad libitum. Rats ate a high fat diet for 6 weeks. Body weight and non-fasting blood glucose levels were measured twice weekly. Non-fasting insulin levels were measured once a week. The procedures for the care, use, and euthanasia of experimental animals followed the protocols and regulations set forth by the Animal Care and Use Committee of the University of Miami and conformed to the Guide for the Care and Use of Laboratory Animals published by the US National Institutes of Health.

### Proteomics

Following incubation in high glucose and free fatty acids or incubation under normoglycemic conditions (control) cells were washed, lysed and proteins extracted in lysis buffer (Cell Signaling). Samples were then concentrated using 3 kDa molecular weight cut off filter (Amicon), and centrifuged for 18 min at 4000×*g* at 4 °C. Protein concentration was determined using the Bradford assay. Up to 50 µg of protein was prepped using the filter aided sample preparation method (Expedon). Samples were processed for proteomic analysis. Specifically, 200 μl of 8 M Urea and 10 mM DTT (Sigma) were added to each sample for reduction. Samples were vortexed for 30 min at room temperature. Samples were then transferred to FASP spin filters and centrifuged for 10 min at 14,000×*g* followed by a subsequent spin after addition of 200 μl of fresh 8 M urea solution (no DTT). Sample alkylation was performed by adding 10 μl re-suspended iodoacetamide (provided by the kit) and incubated at room temperature for 20 min. Samples were centrifuged and washed twice with 100 µl of 8 M urea and once with 100 µl of 50 mM ammonium bicarbonate. For digestion, 2 µg of Sigma trypsin (Sigma Aldrich) was added to each sample. Samples were incubated at 37 °C overnight with gentle linear shaking. Elution of the samples was performed the next day by first adding 40 µl of ammonium bicarbonate prior to centrifugation, and then adding 110 µl of optima water. Samples were then dried down in a speed vacuum for 1.5 h and desalted using Pierce C18 desalting spin columns (Pierce). The desalted samples were dried down and re-suspended in 20 mM ammonium formate.

Stable isotope labeling with the 8-plex iTRAQ reagent (SCIEX, Framingham, MA) and LC–MS/MS was used for peptide identification and quantification. Here, peptides and proteins are assigned abundance ratios relative to a reference sample. To allow for batch to batch comparisons between experiments all samples were compared to a quality control reference sample (QCP) that consisted of aliquots from all samples. The QCP samples are labeled with 113 reagent according to the manufacturer’s recommendation (SCIEX, Framingham, MA). The mixture of samples (~ 5 µg) were then fractionated on an Eksigent 2D NanoLC Ultrasystem coupled to an LTQ Orbitrap Velos mass spectrometer (Thermo Fisher Scientific). The peptides mixtures were injected into a 5 cm SCX column (300 μm ID, 5 μm, PolySULFOETHYL Aspartamide column from PolyLC, Columbia, Md.) with a flow of 4 μl/min and eluted in 10 ion exchange elution segments into a C18 trap column (2.5 cm, 100 μm ID, 5 μm, 300 Å ProteoPep II from New Objective, Woburn, Mass.) and washed for 5 min with H_2_O/0.1% FA. The separation was then further carried out at 300 nl/min using a gradient of 2–45% B [H_2_O/0.1% FA (solvent A) and ACN/0.1% FA (solvent B)] for 120 min on a 15 cm fused silica column (75 μm ID, 5 μm, 300 Å ProteoPep II from New Objective, Woburn, Mass.). Full scan MS spectra (m/z 300–2000) was acquired in the Orbitrap with resolution of 30,000. The most intense ions (up to 10) were sequentially isolated for fragmentation using high energy C-trap dissociation (HCD) and dynamically excluded for 30 s. The resulting fragment ions were then scanned in the orbitrap with resolution of 7500. The LTQ Orbitrap Velos was controlled by Xcalibur 2.1 with foundation 1.0.1.

Peptides and proteins were identified using Proteome Discoverer software (Thermo Electron) with Mascot search engine against SwissProt database. Search parameters included 10 ppm for MS tolerance, 0.02 Da for MS^2^ tolerance, and full trypsin digestion allowing for up to 2 missed cleavages. Carbamidomethylation (C) was set as the fixed modification. Oxidation (M), iTRAQ, and deamidation (NQ) were set as dynamic modifications. Peptides and protein identifications were filtered with Mascot Significant Threshold (p < 0.05). The filters allowed a 99% confidence level of protein identification (1% FDR).

### Immunofluorescence staining and flow cytometry

For immunofluorescence staining, cells were seeded at 50,000 cells/well in 24-well plates or 150,000 cells/dish in a petri dish embedded with a glass slide on the bottom and allowed to attach overnight. On day 2, cells were washed with PBS and fixed with cold acetone/methanol for 1.5 min. Cells were grown, fixed, and stained directly in 24-well plates with or without PDL coated glass slides. Cells were fixed and permeabilized for 10 min on ice with ice cold methanol. The fixative was aspirated and cells were rinsed 3 times in PBS for 5 min each. Cells were blocked in Blocking Buffer for 4 h then incubated in primary antibody (anti-mouse CCDC47 [Thermo Scientific] and anti-rabbit SERCA2 [Abcam]) at 1:500 in Antibody Dilution Buffer overnight at 4 °C. Cells were then rinsed 3 times in PBS for 5 min each and then incubated in fluorochrome-conjugated secondary antibody (goat anti-rabbit IgG Alexa Fluor 488 and goat anti-mouse IgG Alexa Fluor 594) diluted (1:500) in Antibody Dilution Buffer for 2 h at room temperature in dark. Cells were then rinsed in PBS for 3 times, cover-slipped with DAPI, and imaged by fluorescence microscopy.

For detection of CCDC47 expression using flow cytometry, H9C2 cells were stained with Mito-tracker© Red FM for 5 min (Invitrogen), washed, then fixed and permeabilized with 100 μl of cold acetone/methanol (1:1 v/v), shook briefly, and incubated for 90 s. The cells were re-suspended to approximately 10^6^ cells/ml in ice cold PBS with 1% BSA and blocked for 4 h at room temperature. Cells were then incubated in 5 μg/ml of the primary antibody (CCDC47, Thermo Scientific; SERCA2, Abcam) in 1% BSA/PBS overnight at 4 °C in the dark. Cells were then washed 3 times in ice cold PBS followed by centrifugation at 1000×*g* for 5 min prior to incubation in secondary antibody (anti-rabbit IgG FITC for CCDC47 and anti-mouse IgG fluorophore for SERCA-2) diluted in 1% BSA/PBS at 1:400, re-suspended in the solution, and incubated for 2 h at room temperature in the dark. Cells were then washed 3 times by centrifugation at 1000×*g* for 5 min and re-suspended in ice cold PBS. Cells were analyzed immediately on the flow cytometer.

### Calcium assay

H9C2 cells were seeded at 150,000 cells/well in 6-well plates and allowed to attach overnight. On day 2, cells were transfected with pcDNA™3.1 (+) Mammalian Expression Vector (ThermoFisher) containing CCDC47 gene or control sequence. Transfections were performed using TransIT-TKO^®^ Transfection kit (Mirus) according to the manufacturer’s instruction. Cells were assayed 24 h after transfection for calcium homeostasis, and CCDC47 protein levels were increased as shown in Additional file 3: Figure S2. Cytosolic calcium was stained using the FLUOFORTE calcium assay kit (Enzo) per manufacturer’s recommendation. Release of ER calcium to cytosol was induced by a low concentration of ionomycin (3 μM). Fluorescent intensity was measured at 340 nm and 380 nm every 2 s for 4 min to observe cytosolic calcium flow.

### Western blot analysis

Cells and tissues were lysed in Cell Lysis Buffer (Cell Signaling). Protein concentration was quantified using the BCA assay and 10 μg of total protein from each sample were separated on 12% SDS-PAGE gels. Proteins were then transferred onto a PVDF membrane overnight then blocked for 1 h. Membranes were incubated in CCDC47 (Thermo Scientific) primary antibody overnight at 4 °C then and incubated in secondary antibody HRP-conjugated anti-rabbit IgG (Thermo Scientific) for 1 h. Membranes were developed using SuperSignal West Dura Chemiluminescent Substrate (Thermo Scientific) and densities of the immunoreactive bands were quantified by Image J software. After assessment of CCDC47 protein levels, membranes were stripped and probed with β-actin (Abcam) or α-tubulin (Thermo Scientific), used as loading controls, and developed using SuperSignal West Dura Chemiluminescent Substrate.

### Tissue homogenization and RNA isolation

Total RNA was isolated and purified from cells and tissue using TRIzol in accordance to manufacturer’s recommendation (Invitrogen). Briefly, samples were homogenized and incubated in TRIzol for 5 min at room temperature to permit complete dissociation of the nucleoprotein complex. One hundred microliters of chloroform were added per 0.5 ml of TRIzol reagent used for homogenization. The tube was capped securely and shaken vigorously for 15 s prior to incubation for 2 min at room temperature. The samples were then centrifuged at 12,000×*g* for 15 min at 4 °C. The aqueous phase was removed and transferred into a new tube and purified using the RNA Isolation Procedure using RNeasy Mini Kit (Qiagen).

### Reverse transcription and real-time PCR

Five micrograms of total RNA was reversed transcribed using a High Capacity cDNA Reverse Transcription Kit (Applied Biosystems). Two hundred fifty ng were subjected to real-time PCR for mRNA of target genes using a taqman qPCR gene expression assay. Genes were normalized to 18s RNA of each sample (single plex).

### Data analysis

Proteomics data was analyzed after filtering to remove proteins and/or experiments that had an excess of missing data and only proteins that were matched across biological replicates (donor samples) were analyzed. A total of 1283 proteins were captured that were matched across biological samples. For each protein the differential between control and perturbation (treatment with FFA, l-carnitine, and high glucose) was compared and analyzed using Student’s *t* test. The differential protein dataset was then compared to that annotated in the Uniprot Consortium [39, 40]. First the UniProt-GOA database was downloaded into a Microsoft excel spreadsheet and duplicate proteins were removed, resulting in a total of 4277 unique proteins listed in the Uniprot cardiovascular gene ontology annotation (UniProt-GOA) [41]. The differential dataset (912 proteins) was then compared to those currently annotated Uniprot-GOA database to identify unique proteins. All *Homo sapiens* proteins (Uniprot identification [ID], Entry name, protein name, gene name, and gene ontology [GO] ID) that are currently annotated in the Uniprot database (158,091) was downloaded onto a Microsoft excel sheet and GO IDs were extracted for each unique protein [39, 40]. Calcium regulating and ER localized proteins were then identified and extracted by GO IDs.

To determine the degree of co-localization correlation analysis of overlapping fluoresent intensities of CCDC47 and either mito-tracker or SERCA2 was performed on flow cytometry data using software. Western blot densitometry was performed using NIH ImageJ. Statistical analysis of all data with comparisons between 2 groups were performed using Student’s *t* test and for > 3 using one-way analysis of variance (ANOVA) (GraphPad Prism). A p-value of 0.05 was deemed significant.

## Additional files

**Additional file 1: Figure S1.** In vitro model of cardiomyopathy does not significantly capture proteins from renal disease database. Percentage of proteins in Uniprot’s renal disease database that were captured from the in vitro model.

**Additional file 2: Table S1.** List of Unique proteins associated with Calcium regulation and/or endoplasmic reticulum/sarcoplasmic reticulum (ER/SR).

**Additional file 3: Figure S2.** CCDC47 overexpression in rat cardiomyocytes. Representative Western blot (**A**) and quantification (**B**) of CCDC47 protein in H9C2 cells transfected with empty vector (control) and CCDC47 plasmid. CCDC47 immunoreactivity band density was normalized to β-actin and data are expressed as fold change over control. Data represents n = 3 independent experiments. *p < 0.05 compared to empty vector group of the same time point.

## Abbreviations

ANP: atrial natriuretic peptide
BNP: brain natriurectic peptide
β-MHC: myosin heave chain beta
Ca^2+^: calcium
CAD: coronary artery disease
CRAC: Ca^2+^ release-activated Ca^2+^ channel
CVD: cardiovascular disease
DCM: diabetic cardiomyopathy
DIO: diet induced obese/obesity
ER: endoplasmic reticulum
ECC: excitation–contraction coupling
GO: gene ontology
ID: identification
NCX: sarcolemmal Na/Ca^2+^ exchange
O-GlcNAc: O-linked *N*-acetylglucosamine
PLN: phospholamban
RyR: ryanodine receptor
SR: sarcoplasmic reticulum
SERCA: SR Ca^2+^ ATPase
SOCE: store-operated Ca^2+^ entry
UPR: unfolded protein response
