# Supplementary figures and images for: Dysregulation of the calcium handling protein, CCDC47, is associated with diabetic cardiomyopathy
Source: Cell Biosci. 2018 Aug 17;8:45. doi: 10.1186/s13578-018-0244-0 (PMC6098598; doi:10.1186/s13578-018-0244-0)

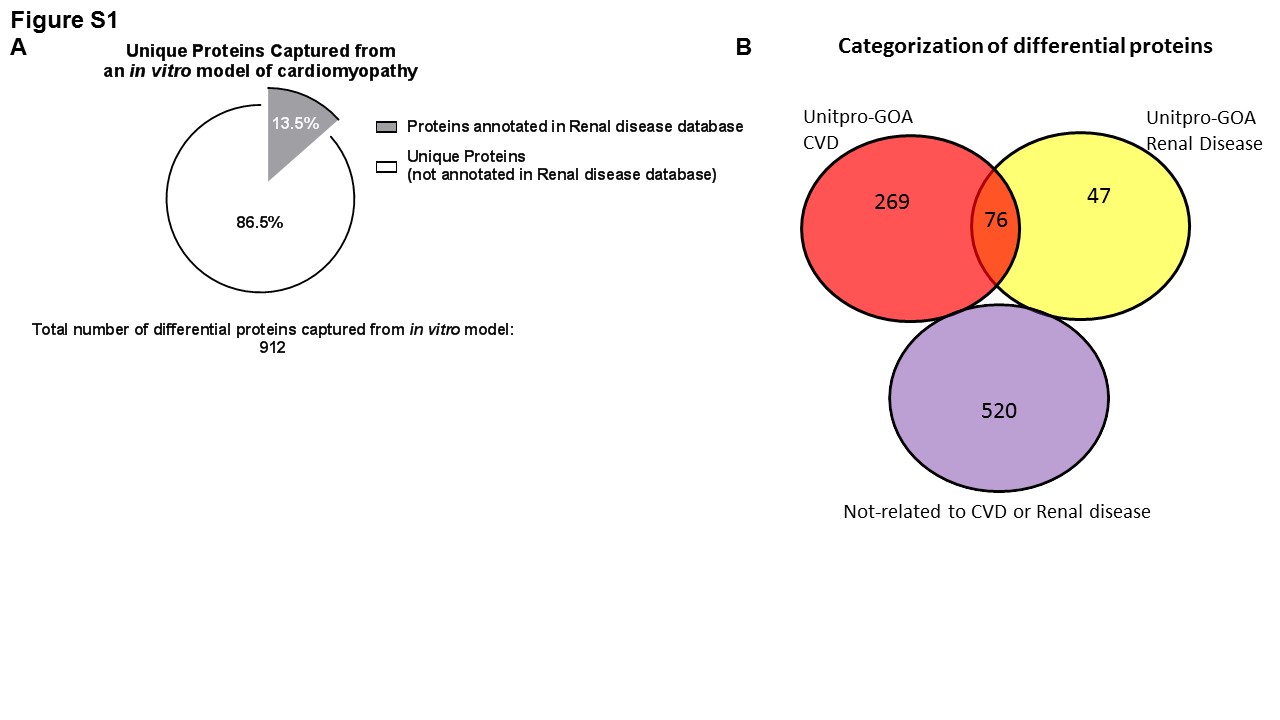

Supplement: Supplementary file 1 — Additional file 1: Figure S1. In vitro model of cardiomyopathy does not significantly capture proteins from renal disease database. Percentage of proteins in Uniprot’s renal disease database that were captured from the in vitro model. [file 13578_2018_244_MOESM1_ESM.jpg]

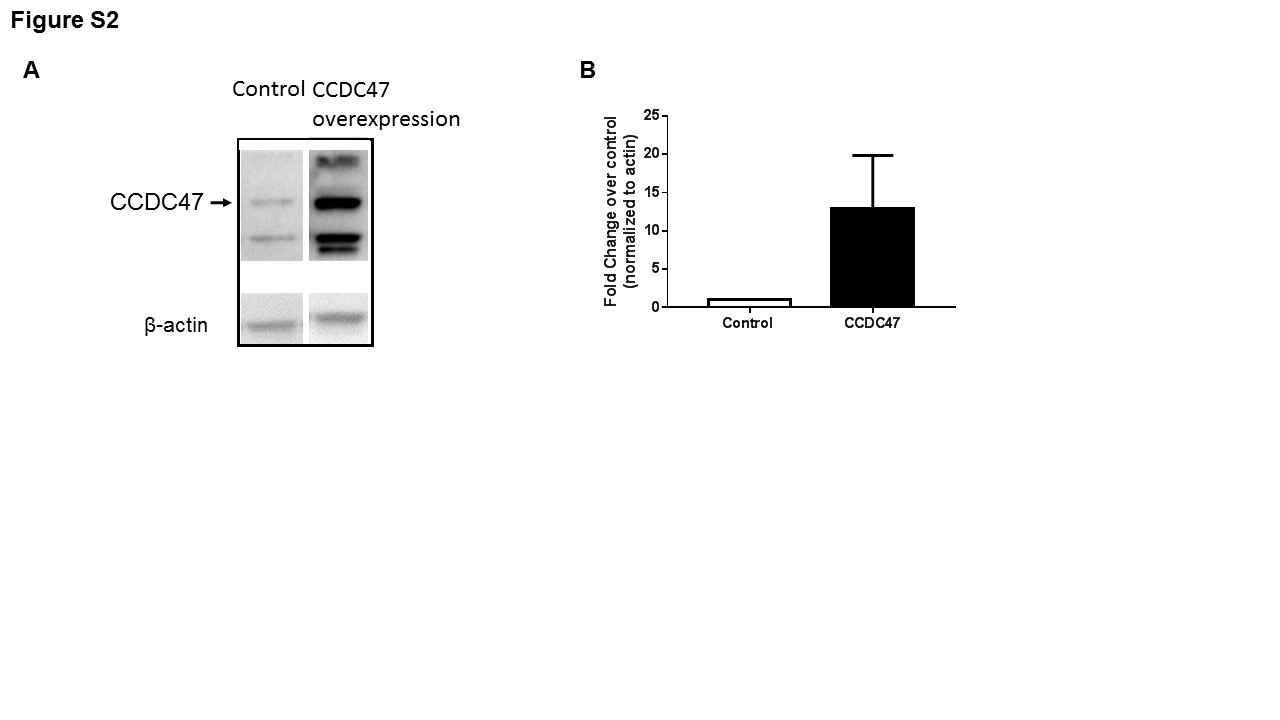

Supplement: Supplementary file 3 — Additional file 3: Figure S2. CCDC47 overexpression in rat cardiomyocytes. Representative Western blot (A) and quantification (B) of CCDC47 protein in H9C2 cells transfected with empty vector (control) and CCDC47 plasmid. CCDC47 immunoreactivity band density was normalized to β-actin and data are expressed as fold change over control. Data represents n = 3 independent experiments. *p < 0.05 compared to empty vector group of the same time point. [file 13578_2018_244_MOESM3_ESM.jpg]
